# First Complete Genome Sequence of *Arracacha virus A* Isolated from a 38-Year-Old Sample from Peru

**DOI:** 10.1128/genomeA.00141-17

**Published:** 2017-05-04

**Authors:** Ian P. Adams, Neil Boonham, Roger A. C. Jones

**Affiliations:** a Fera, Sand Hutton, York, United Kingdom; b Institute for Agrifood Research Innovations, Newcastle University, Newcastle upon Tyne, United Kingdom; c Institute of Agriculture, Faculty of Science, University of Western Australia, Crawley, Western Australia, Australia; d Department of Agriculture and Food Western Australia, South Perth, Western Australia, Australia

## Abstract

We present here the first complete genomic sequence of *Arracacha virus A* from a Peruvian arracacha sample collected in 1975 and compare it with the genomes of other nepoviruses. Its RNA1 and RNA2 both had greatest amino acid identities with those of the subgroup A nepovirus *Melon mild mottle virus*.

## GENOME ANNOUNCEMENT

In 1975, during a virus survey of subsistence plantings containing crop mixtures at Umari, Huanuco Department, in the Peruvian Andes, a virus was isolated from arracacha plants [Peruvian parsnip (*Arracaccia xanthorhiza*), family Apiaceae] showing pronounced leaf mosaic symptoms. The virus was mechanically transmissible to 38 species from 10 plant families. Following characterization using electron microscopy, density centrifugation, and serology, it was identified as a member of the *Nepovirus* genus and named *Arracacha virus A* (AVA) (1, 2). In 1978, AVA-infected leaf samples were dried over calcium chloride in Peru, sealed in glass vials, and sent to the United Kingdom. They currently form part of what is now called the Fera plant virus collection.

In 2015, total RNA was extracted from the freeze-dried AVA-infected leaf material using an RNeasy kit (Qiagen, United Kingdom), including the optional DNase treatment. An indexed plant ribosome-subtracted sequencing library was then produced from the total RNA using the ScriptSeq complete plant leaf kit (Illumina, USA), according to the manufacturer’s instructions. The indexed library was then sequenced along with others on a MiSeq instrument (Illumina), using a 600-cycle V3 kit. The resulting 492,656 paired reads were trimmed on the 3′ end to a quality score of 20 using Sickle (3), assembled using Trinity (4), and the resulting contigs compared to the GenBank nr and nt databases using BLAST+ (5). Reads of viral origin were extracted using MeganN (6). Two contigs of 7,411 and 3,852 nucleotides (nt) in length were assembled and shown by similarity to other nepoviruses to represent the RNA1 and RNA2 components of the AVA genome, respectively.

The AVA RNA1 encoded a putative 266-kDa polyprotein with an amino acid identity resembling those of the subgroup A nepoviruses *Melon mild mottle virus* (39%) (7) and *Grapevine fanleaf virus* (36%) (8). The AVA RNA2 encoded a putative 127-kDa polyprotein with 32% and 30% amino acid identities to *Melon mild mottle virus* and *Potato black ringspot virus* (9), another subgroup A nepovirus, respectively. These findings, along with the absence of any significant homology between the 3′ untranslated regions (UTRs) of the RNA1 and RNA2 molecules confirm that AVA belongs to subgroup A of the *Nepovirus* genus.

### Accession number(s).

The sequences were deposited in GenBank under accession numbers KY569301 (RNA1) and KY569302 (RNA2).
